# Multiple Extramedullary Plasmacytoma with Lytic Bony Lesions: A Rare Case Report

**DOI:** 10.1155/2013/291359

**Published:** 2013-02-12

**Authors:** Akhlak Hussain, Mohinder Singh, Kuldip Singh, Harjot Bagga

**Affiliations:** ^1^Department of Surgery, Rajindra Hospital, Patiala 147001, India; ^2^Department of Radiotherapy, Rajindra Hospital, Patiala 147001, India

## Abstract

*Objective*. Multiple extramedullary plasmacytoma lesions involving subcutaneous tissue, breast, mediastinal tissue, spleen, and soft tissue of pelvic region along with multiple bones plasmacytomas without marrow plasmacytosis are a very rare presentation. *Design*. Case report. *Result*. A 54-year-old female was found to have multiple small bony lytic lesions, multiple extramedullary soft tissue plasmacytomas, serum M protein >3 g/dL, and elevated ESR. Bone marrow aspirate did not reveal any evidence of multiple myeloma/plasmacytosis. There was no anemia, hypercalcemia, or renal insufficiency. *Conclusion*. Extramedullary plasmacytoma can involve multiple organs at a time including bones and soft tissue without involving bone marrow.

## 1. Introduction

The classical triad of multiple myeloma is marrow plasmacytosis (>10%), lytic bony lesions, and a serum and/or urine M component. Solitary bone plasmacytoma and extramedullary plasmacytoma are among the variants of multiple myeloma. These lesions are associated with an M component in <30% of cases, they may affect younger individuals, and both are associated with median survivals of ≥10 years. Solitary bone plasmacytoma is single lytic bone lesion without marrow plasmacytosis. Extramedullary plasmacytoma usually involves the submucosal lymphoid tissue of nasopharynx or paranasal sinuses without marrow plasmacytosis. Both tumors are highly responsive to local radiation therapy. If M component is present, it should disappear after treatment [1]. 

We are presenting an atypical case having multiple extramedullary plasmacytoma lesions along with multiple bones plasmacytomas without marrow plasmacytosis.

## 2. Case Report

**Figure 1:**
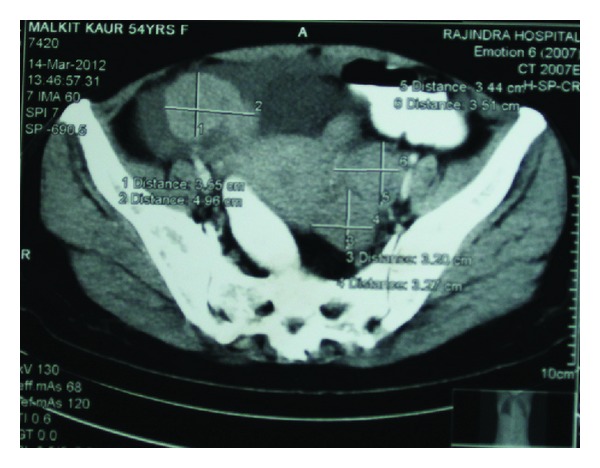
CT showing multiple heterogeneously enhancing masses in the pelvic around the uterus.

**Figure 2:**
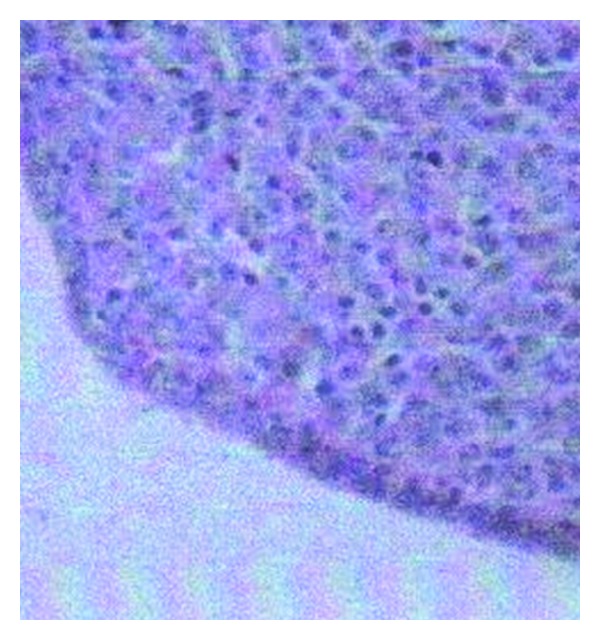
HPE of breast excisional biopsy showing sheets of large nucleolated and plasmacytoid cells permeating breast parenchyma.

**Figure 3:**
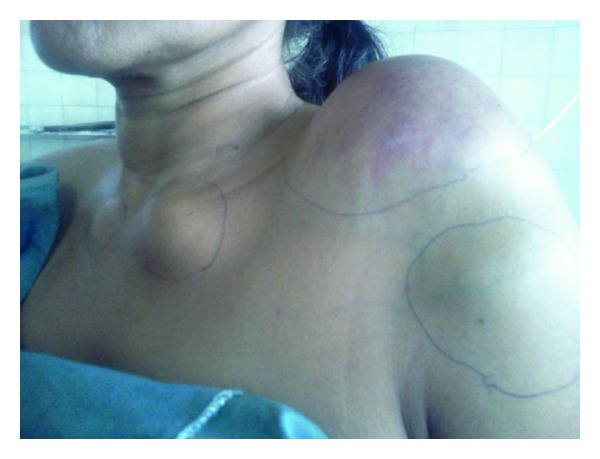
Patient with swelling over left shoulder, bilateral sternoclavicular joints, and left upper arm (before chemotherapy).

**Figure 4:**
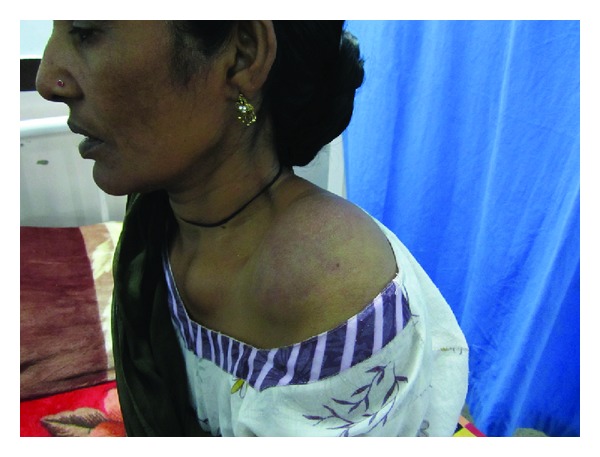
Patient with swellings subsided after 2 cycles of chemotherapy.

A 54-year-old married female had presented with chief complaint of insidious onset of mild pain in left hypochondrium since 3 months, nonradiating, nonshifting, nonreferring, no relation with feeding or exertion, and temporarily relieved by medication. It was associated with feeling of fullness in left upper quadrant of abdomen. There was no history of nausea, vomiting, fever, constipation, or diarrhea. After 10 days she noticed a small swelling on the left shoulder increasing progressively to present size of about 8 × 10 cm, firm, nontender but with redness of overlying skin. After about a week, she noticed two more swellings on both sternoclavicular joint regions, increasing slowly to present size of 3 × 2 cm (left) and 2 × 1 cm (right), firm, nontender without any skin changes. Simultaneously, she found multiple nontender swellings in both breasts, the largest being 4 × 2 cm in the right breast and 2 × 1 cm in the left breast. There was no significant past, personal, and family history. There is history of loss of weight about 10 kg in 3 months and loss of appetite. Her build and general physical examination were normal. There was no positive finding on systemic examination. Blood investigations revealed higher total proteins (10.40 g/dL) and gamma globulin (6.14 g/dL). A : G ratio was decreased (0.41). Albumin level decreased (3.04 g/dL). Serum protein electrophoresis revealed “M” spike in gamma globulin region (56.12% = 5.84 g/dL). Haemoglobin, leucocytes, and platelets levels were normal. Serum electrolytes and renal function tests were normal. Peripheral smear revealed mild microcytosis only. Serum calcium was normal. ESR was increased (106 mm at 1st hour). Serum *β*-2 microglobulin level was increased significantly (3007.60 *μ*g/l). Urine for Bence-Jones protein was negative. U/S revealed 5.3 × 5.2 cm anechoic lesion with multiple internal echoes with few solid components within seen in upper pole of spleen. Multiple hypoechoic masses were seen along bilateral iliac vessels. CT scan showed a large enhancing soft tissue attenuation mass, 8.85 × 6.07 cm, around the left shoulder region and in the left supraclavicular region. Another small soft tissue attenuation mass was seen around left sternoclavicular joint. Nodular pleural thickening with maximum thickness of 2.67 cm was seen on the left side. Left-sided pleural effusion with underlying collapse of the left lung was seen. Right lung field was clear. A soft tissue attenuation mass is seen in the retrosternal region with maximum width of 2.11 cm, lying anterior to the aortic arch with preserved fat planes. Rounded soft tissue density mass was seen in the right breast. Multiple heterogeneously enhancing masses were seen in the pelvis around the uterus (Figure 1). Mass on the right lying anterior to external iliac vessels measures 6.01 × 4.9 cm^2^. Right ovary was not separately defined from the mass. Multiple masses were seen in the left adnexal region, the largest measuring 3.4 × 3.5 cm lying medial to internal iliac vessels. Left ovary was not separately defined. Left-sided psoas muscle was swollen with maximum thickness of 2.58 cm. A well-defined 6.2 × 5.13 cm, cystic mass with solid components and internal septae is seen in the spleen. Other abdominal and thoracic organs were normal. Fluid was present in Morrison's pouch and pelvis. Small lytic lesions are seen in vertebral bodies of spine, ribs, and pelvic bones. Bone marrow aspiration was done twice but did not reveal any abnormality. FNAC of all external swellings was consistent with infiltration of plasma cells with prominent nucleoli, with some of them having two or three nuclei. Incisional biopsy of breast lump correlated well with plasmacytoma (Figure 2). Immunohistochemistry revealed CD45(LCA) and CD138 with immunoreactive score 4+. Combination chemotherapy was started with melphalan, dexamethasone, and thalidomide. The patient has shown a very good response to chemotherapy even after 2 cycles (Figures 3 and 4). A total of 12 cycles were given. Currently the patient is maintaining well.

## 3. Discussion

Plasmacytoma, a neoplastic proliferation of plasma cells, is one form of plasma cell dyscrasia. Plasmacytoma may be primary or secondary to disseminated multiple myeloma and may arise from osseous (medullary) or nonosseous (extramedullary) sites. Primary extramedullary plasmacytoma can be solitary or multiple [2]. The International Myeloma Working Group in 2003 recognized a separate classification of plasmacytomas that occur as multiple sites of disease in soft tissue, bone, or both soft tissue and bone as multiple solitary plasmacytoma [3].

In a review of more than 400 published articles, 82.2% of extramedullary plasmacytomas were found in the upper aerodigestive tract with 17.8% arising in the gastrointestinal tract, urogenital tract, skin, lung, and breast in that order [2]. Although liver, spleen, and lymph nodes are common extramedullary manifestations of multiple myeloma, primary extramedullary plasmacytomas of these organs—including the pancreas and adrenal gland—are extremely rare.

Multiple solitary plasmacytomas, which may be recurrent, occur in up to 5% of patients with an apparently solitary plasmacytoma and may involve soft tissue or bone. Diagnostic criteria include absent or low serum or urinary level of monoclonal immunoglobulin, more than one localized area of bone destruction or extramedullar tumor of clonal plasma cells which may be recurrent, normal bone marrow, normal skeletal survey, MRI of spine and pelvis, and no related organ or tissue impairment [4].

Leake presented a case of plasmacytoma of the pancreas. A blood film showing rouleaux formation and a skeletal survey demonstrating multiple lytic lesions confirmed multiple myeloma [5]. In the presenting case bone marrow was not involved. Tüting andBork had reported a case of a solitary primary cutaneous plasmacytoma developed on the left thigh without bone marrow involvement [6]. Wong et al. presented two cases with a slowly growing, painless, and solitary mass on the chest wall. Histologically, one case was composed of mature-looking plasma cells, while the other was composed of immature and anaplastic plasma cells, infiltrating the dermis. The epidermis was spared. There was no evidence of marrow disease even on repeated marrow biopsies, although extracutaneous lesions were detected in one patient [7]. Kaviani et al. presented a 70-year-old woman with bilateral breast masses who underwent excisional biopsy for suspected primary carcinoma that subsequently proved to be a recurrence from extramedullary plasmacytoma of the mediastinum for which she got treatment 5 years back [8]. Shahid et al. reported a rare case of solitary lytic lesion in the medial part of clavicle with biopsy revealing plasmacytoma [9].

Main findings of our case are

- multiple small lytic lesions in vertebral bodies, ribs, and pelvic bones,
- multiple extramedullary plasmacytomas involving subcutaneous tissue, bilateral breasts, spleen, and pelvic cavity,
- serum *β*-2 microglobulin <3.5 mg/L, serum M protein >3 g/dL, and elevated ESR,
- bone marrow aspirate showing no evidence of multiple myeloma/plasmacytosis,
- no anemia, hypercalcemia, or renal insufficiency,
- no Bence-Jones protein in urine.

These findings seem to be a combination of multiple extramedullary plasmacytomas and multiple bones plasmacytomas. The usual presentation of both these neoplasms is solitary, that is, include single organ [1]. In our case, there are multiple lesions which are a rare presentation.

In summary, it can be concluded that plasmacytoma can involve multiple organs at a time including bones and soft tissue without involving bone marrow.
